# First person – Jacqueline Simonet

**DOI:** 10.1242/bio.054718

**Published:** 2020-08-13

## Abstract

First Person is a series of interviews with the first authors of a selection of papers published in Biology Open, helping early-career researchers promote themselves alongside their papers. Jacqueline Simonet is first author on ‘CTP synthase polymerization in germline cells of the developing *Drosophila* egg supports egg production’, published in BiO. Jacqueline conducted the research described in this article while a postdoctoral research associate in Jeffrey Peterson's lab at Fox Chase Cancer Center, Philadelphia, PA and is now a Visiting Assistant Professor at Arcadia University, PA, investigating the regulation of cell metabolism, particularly the regulation of biosynthetic enzymes by filamentation using *Drosophila* as a model system.


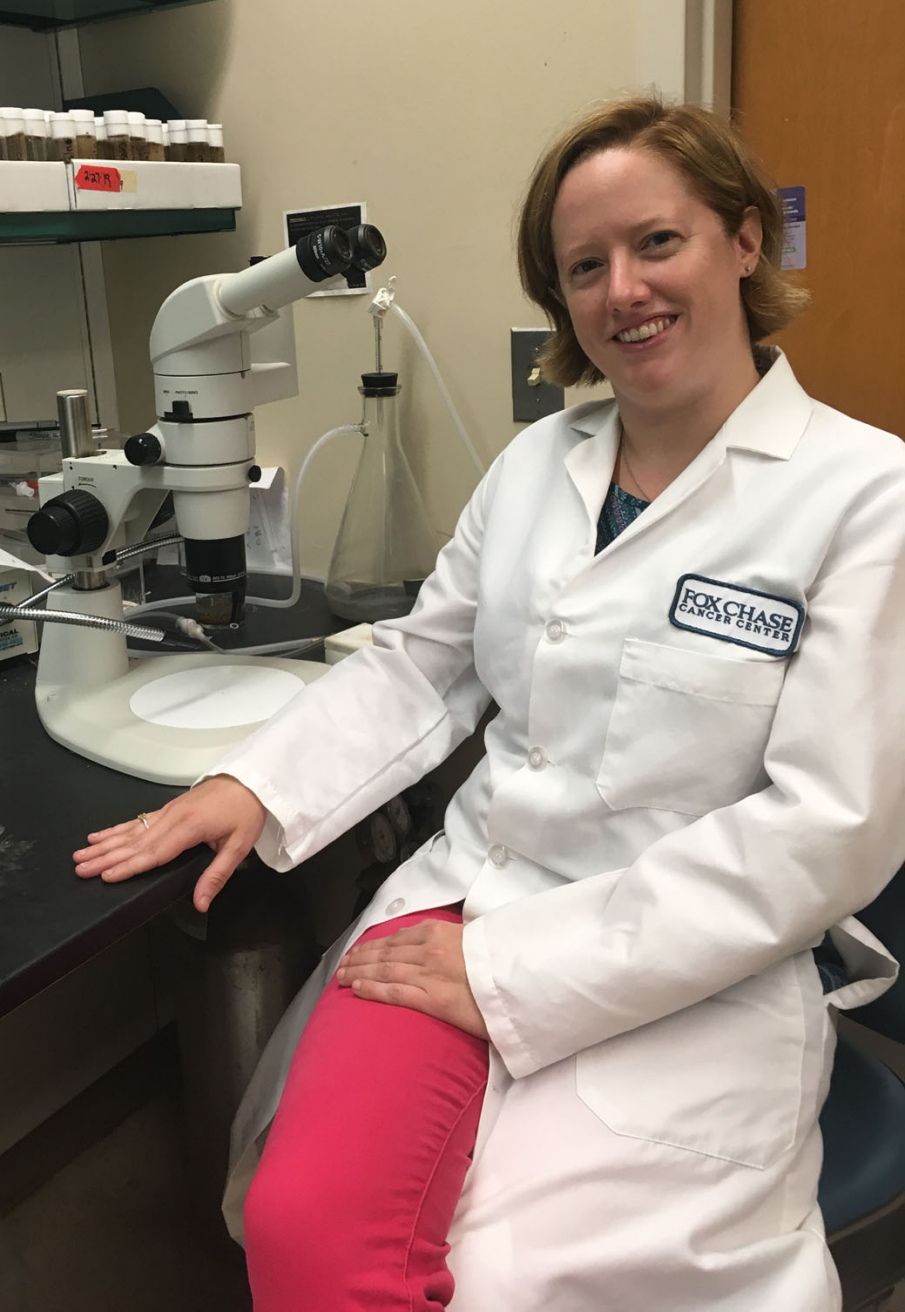


**Jacqueline Simonet**

**What is your scientific background and the general focus of your lab?**

My background is in developmental biology, cell and molecular biology, and neurobiology. The general focus of my lab is basic cell metabolism using *Drosophila* as a model system.

**How would you explain the main findings of your paper to non-scientific family and friends?**

We study an enzyme, called CTP synthase, whose job is to make one of the building blocks of DNA. This enzyme forms large structures called filaments. These filament structures appear to be important for the normal function of this enzyme, at least in some cell types, because when we removed them in fruit fly ovaries the flies couldn't make as many eggs.

**What are the potential implications of these results for your field of research?**

These filaments have been studied for many years but there are very few examples of their function in normal biological systems. Creating this model in *Drosophila* will hopefully allow us to study the function and regulation of these filaments *in vivo*.

**What has surprised you the most while conducting your research?**

I think the thing that was the most surprising was that the loss of CTPS filaments in the *Drosophila* germ cells only results in a decrease in fertility not a complete loss. I would have thought that if they are forming these large filaments that it would be necessary for egg development, but it seems like with enough functional CTPS egg development can still proceed, but at a lower level than with filaments.

“…the loss of CTPS filaments in the *Drosophila* germ cells only results in a decrease in fertility not a complete loss…”

**What, in your opinion, are some of the greatest achievements in your field and how has this influenced your research?**

The discovery by Chalongrat Noree et al. that CTPS forms filaments in yeast and *Drosophila* was obviously extremely important for starting this field. As was the discovery of these filaments in bacteria by Michael Ingerson-Mahar et al. and the discovery of these filaments in the rods and rings in human cells by Wendy C. Carcamo et al..

**What changes do you think could improve the professional lives of early-career scientists?**

I think that there should be more mechanisms for funding scientists who want to continue to work in laboratories as bench researchers, such as research associates. There is a lot of funding put into training graduate students and postdoctoral associates to do great science at the bench and then they have to leave the lab to write grants or find jobs outside of academia. A lot of technical and scientific knowledge is lost from laboratories and the community as a whole and it can continue to push those with the most knowledge and expertise in the lab out into the offices and away from academia in general.

**Figure:**
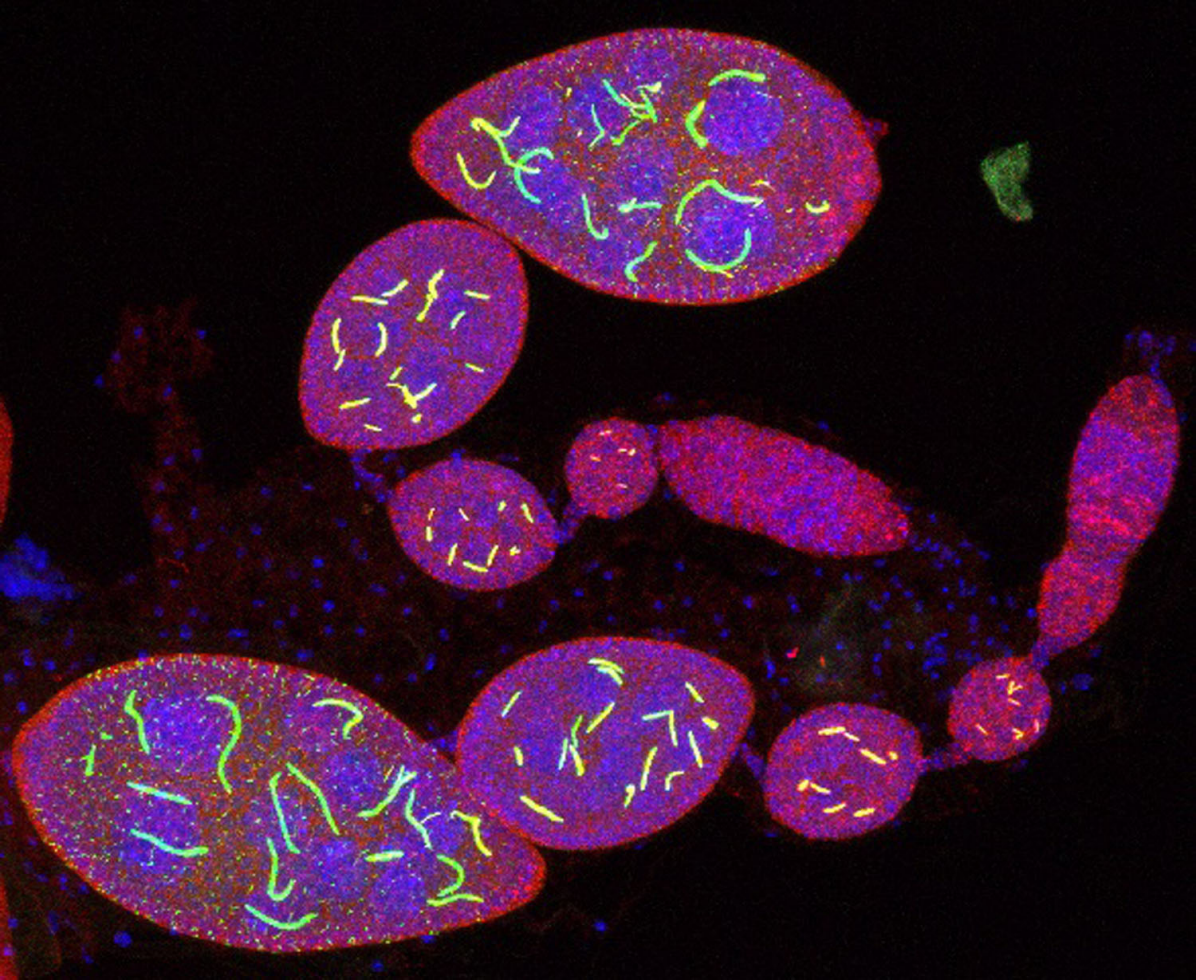
**A Z-projection confocal image of GFP-CTPS and Flag-tagged CTPS stained with an antibody form filaments together in *Drosophila* ovaries, which are also stained with DAPI to show the nuclei.**

“There is a lot of funding put into training graduate students and postdoctoral associates to do great science at the bench and then they have to leave the lab to write grants or find jobs outside of academia.”

**What's next for you?**

Hopefully continuing to teach at Arcadia University and continue this research with the undergraduates here.
